# Impact of CPAP on physical exercise tolerance and sympathetic-vagal balance in patients with chronic heart failure

**DOI:** 10.1590/bjpt-rbf.2014.0037

**Published:** 2014

**Authors:** Hugo V. Reis, Audrey Borghi-Silva, Aparecida M. Catai, Michel S. Reis

**Affiliations:** 1 Research Group in Cardiorespiratory Physical Therapy (GECARE), Department of Physical Therapy, Faculty of Medicine, Universidade Federal do Rio de Janeiro (UFRJ), Rio de Janeiro, RJ, Brazil; 2 Laboratory of Cardiopulmonary Physical Therapy, Department of Physical Therapy, Universidade Federal de São Carlos (UFSCar), São Carlos, SP, Brazil; 3 Laboratory of Cardiovascular Physical Therapy, Department of Physical Therapy, UFSCar, São Carlos, SP, Brazil

**Keywords:** non-invasive ventilation, heart rate variability, chronic heart failure, exercise tolerance, continuous positive airway pressure, physical therapy

## Abstract

**Background:**

Chronic heart failure (CHF) leads to exercise intolerance. However, non-invasive ventilation is able to improve functional capacity of patients with CHF.

**Objectives:**

The aim of this study was to evaluate the effectiveness of continuous positive airway pressure (CPAP) on physical exercise tolerance and heart rate variability (HRV) in patients with CHF.

**Method:**

: Seven men with CHF (62±8 years) and left ventricle ejection fraction of 41±8% were submitted to an incremental symptom-limited exercise test (IT) on the cicloergometer. On separate days, patients were randomized to perform four constant work rate exercise tests to maximal tolerance with and without CPAP (5 cmH_2_O) in the following conditions: i) at 50% of peak work rate of IT; and ii) at 75% of peak work rate of IT. At rest and during these conditions, instantaneous heart rate (HR) was recorded using a cardiofrequencimeter and HRV was analyzed in time domain (SDNN and RMSSD indexes). For statistical procedures, Wilcoxon test or Kruskall-Wallis test with Dunn's post-hoc were used accordingly. In addition, categorical variables were analysed through Fischer's test (p<0.05).

**Results:**

There were significant improvements in exercise tolerance at 75% of peak work rate of IT with CPAP (405±52 vs. 438±58 s). RMSSD indexes were lower during exercise tests compared to CPAP at rest and with 50% of peak work rate of IT.

**Conclusion:**

These data suggest that CPAP appears to be a useful strategy to improve functional capacity in patients with CHF. However, the positive impact of CPAP did not generate significant changes in the HRV during physical exercises.

## Introduction

Patients with chronic heart failure (CHF) have reduced exercise tolerance as the main outcome of the disease^1^. Reduced exercise tolerance is due to cardiac conditions but also to respiratory and appendicular muscle dysfunction^1^. Several factors such as chronic hypoxia, oxidative stress, nutritional depletion, peripheral muscle disuse, medication effects and sympathetic-vagal imbalance^2^ are important contributors. Therefore, reduction in exercise tolerance could be attributed to a redistribution of blood flow to the respiratory muscles as a result of work demand and metabolites in the muscles. The consequence would be greater sympathetic vasoconstrictor response in the peripheral muscles, whose metabolic efficiency is compromised by lower blood flow^1^.

The use of non-invasive ventilation (NIV) has had its efficacy proven in various conditions of acute respiratory failure, particularly as a treatment option for the management of patients with acute pulmonary edema^3^. Positive pressure decreases the pulmonary shunt through expansion of collapsed alveoli, thereby improving gas exchange and oxygenation of the tissues^4^. The NIV also reduces the transmural pressure of the left ventricle, the afterload, and hence improves cardiac output^5^. Furthermore, the use of NIV with a continuous pressure level improves functional residual capacity and lung compliance, generating lower ventilatory work^6^, which may represent an important effect for those patients who have respiratory muscle fatigue during exercise^7^. Few studies^7^ ^-^ ^10^ have evaluated the response of this therapeutic approach in the improvement of physical exercise performance of patients with CHF. In addition, some of the studies^8^ ^-^ ^10^ only evaluated the impact of rest on exercise tolerance and not the combined effect of NIV and physical exercise. Knowledge of the efficacy of NIV, as a supporting therapeutic approach in the prescription of physical exercises for patients with CHF, is particularly important in the area of cardiovascular therapy. These patients have reduced exercise tolerance and the peripheral musculature^1^ is the target of physical therapy interventions.

However, it is important to emphasize that the application of NIV is able to cause important hemodynamic repercussions, especially when prescribed inadvertently. Therefore, studies have shown the importance of heart rate variability (HRV), which reflects the sympathetic-vagal balance of the sinus node, for understanding the cardiovascular adjustments during application of NIV at rest in patients with chronic heart failure^11^ ^-^ ^17^. However, little is known about HRV when physical activity is associated with the application of NIV.

In this context, the objective of this study was to evaluate whether the use of NIV through a continuous pressure level (CPAP) in a protocol of physical exercise at constant load is able to improve tolerance to physical exercise and to determine if the CPAP could influence the sympathetic-vagal modulation in physical exercise in patients with CHF.

## Method

### Sample

Seven male patients with a clinical diagnosis of CHF volunteered to participate in this cross-sectional study. Subjects were recruited from a public health primary care facility and to be included in the study presented with the following characteristics: previous history of CHF caused by systolic left ventricular dysfunction documented in the last six months (ejection fraction of the left ventricle <45%), clinically stable in the last three months and no history of angina or pulmonary disease. Subjects with clinical and/or functional evidence of chronic lung disease (FEV_1_/FVC <70%)^18^, with exercise induced asthma, angina or significant arrhythmias, myocardial infarction within the last six months and those who had participated in a cardiovascular rehabilitation program in the year preceding the study, were excluded. All subjects were submitted to clinical evaluation and pulmonary function tests, assessment of functional capacity according to the New York Heart Association (NYHA)^19^, biochemical tests, ECG and a symptom-limited physical exercise test prior to the study. In addition, subjects with CHF had their medication optimized. All subjects signed an informed consent and the protocol was approved by the Ethics Committee from Universidade Federal de São Carlos (UFSCar), São Carlos, SP, Brazil (protocol 238/06).

### Experimental protocol

The research was conducted in an acclimatized laboratory at temperatures between 22 ºC and 24 ºC and relative humidity between 50% and 60%, during the same period of the day (between 8h and 12h). Prior to and on the day of the test, each subject was asked to ensure they avoided consumption of stimulating beverages, avoided physical activity for 24 hours prior to the tests; consumed light meals and slept for at least 8 hours.

Initially, the subjects were familiarized with the experimental equipment and environment and the researchers involved in the study. Prior to the tests, the subjects were evaluated and examined to ensure that the directions given had been strictly followed. In addition, systolic and diastolic blood pressure, lung auscultation and SpO_2_ were assessed.

### Pulmonary function

Spirometry was performed using a Vitalograph^(r)^ spirometer (Hand-Held 2021 instrument. Ennis, Ireland). The CVF test was conducted to determine the FEV1 and FEV1/CVF ratio. The reference values used were those suggested by Knudson et al.^20^ and were expressed in terms of BTPS (*Body Temperature Pressure Standard*). Technical procedures, criteria for acceptability and reproducibility, were performed according to the guidelines recommended by the *American Thoracic Society* ^21^.

### Incremental physical exercise protocol

The assessment was performed by a cardiologist to determine the maximum load the patients were able to achieve. In addition, this step was considered important to assess the clinical and functional conditions of the cardiovascular and peripheral muscular systems of the subjects and to identify evidence of cardiorespiratory comorbidities elicited by physical exercise. Initially, the subjects were evaluated using an ECG with the standard 12-lead ECG, followed by the evaluation of the electrocardiographic signal from the derivations MC5, DII modified and V2 in the following conditions: supine, sitting, apnea (15 s) and hyperventilated (15 s). The exercise test was performed on a cycloergometer with electromagnetic brakes (Quinton 400 Corival Ergometer, Croningen, Netherlands) and power increments externally controlled by a microprocessor model *Workload Program* (*Quinton*, *Croningen, Netherlands)*. The subjects remained seated with the knees flexed at 5-10º. Initially, a 2-minute warm up period was performed with no load, corresponding to 4 watts (W). Following the warm up, the subjects performed, increments of 5W every 3 minutes at 60 rpm, until physical exhaustion or it was impossible for them to maintain the pedaling speed. The test was stopped on the first indications of signs and/or symptoms such as dizziness, nausea, cyanosis, complex arrhythmias, excessive sweating, angina and peripheral oxygen desaturation. During the test, subjects were monitored from the MC5 derivation, DII modified and V2. The measurements of HR, blood pressure (auscultation method) and electrocardiographic recordings were performed in the 30 final seconds of each power level and at the 1^st^, 3^rd^, 6^th^ and 9^th^ minutes of recovery. At the end of the recovery period, with the subject in the supine position, the standard 12-lead ECG was performed. In addition to the variables described above, using formulae recommended by the *American Heart Association* (which take into account peak load and body mass), maximum oxygen consumption (VO_2peak_) achieved by the subjects was obtained. Throughout the test, peripheral oxygen saturation (SpO_2_) was measured using pulse oximetry (*Oxyfast, Takaoka*, Brazil).

### Protocol of constant load in spontaneous breath and during CPAP application

All subjects performed four physical exercise tests at a constant load. The order of the tests was randomized by lottery using sealed, opaque numbered envelopes. The tests were performed on two days (two tests per day) with an interval of 48 hours between the days. For the implementation of this protocol, initially, the subjects were kept at rest in the sitting position for about 10 minutes, with the goal to achieve basal values for HR. At the same time, the instantaneous HR was obtained at rest in the sitting position for 15 minutes. Subsequently, subjects were randomized by lottery to perform submaximal exercise at a constant load until maximum tolerance, with and without application of continuous positive airway pressure (CPAP - 5 cmH_2_O, *Breas PV101, Sweden*) using a nasal mask (Respironics, *Murrysville, PA*) under the following conditions: i) 50% of the peak load of the incremental test and ii) 75% of the peak load of the incremental test. The subjects were positioned in the horizontal electronically braked cycle ergometer (Quinton 400 Corival Ergometer, Croningen, Netherlands) with knees flexed between 5º and 10º. Initially, the subjects remained seated on the cycle ergometer at rest for 1 minute and then were instructed to pedal at a cadence of 60 rpm until maximum tolerance. SpO_2_ (*Oxyfast, Takaoka*, Brazil) and ECG (*Ecafix* 500, São Paulo, Brazil), in leads MC5, DII modified and V2, were monitored continuously throughout the experimental protocol. Blood pressure and the modified BORG scale (CR-10) were verified every two minutes with care to avoid interference in the collection of the variables. The constant workload tests were performed on a single day and at the same time to avoid circadian influenceswith an interval of 30 minutes or until cardiovascular variables returned to baseline values. A team of trained researchers conducted the tests and carefully monitored the signs and/or symptoms of exercise intolerance and who could determine when to immediately stop the test.

### Heart rate and R-R intervals

The HR and R-R intervals (R-Ri) were collected, beat-by-beat, using a heart rate monitor (Polar^(r)^ S810i). Data collection occurred during the 15-min rest in the sitting position and during exercise at constant loads at random conditions of 50% and 75% with and without CPAP. The heart rate monitor had a sampling frequency of 1,000 Hz. It was fixed using an elastic belt to the lower third of the sternum and data was simultaneously transmitted to and stored in a watch. Subsequently, through a serial port interface of an infrared sensor, the data were transferred and stored in a computer (Pentium III, 1100 MHz) to be analyzed. The transition points of the protocol were also properly marked for proper data analysis.

### Data analysis

The maximum time for completion of the physical exercise during the protocol of constant workload was identified by tolerance time. The HR, subjective sensation of effort for dyspnea, and discomfort of the lower limb variables were assessed at baseline and at the peak of the protocol. HRV was analyzed in time domain through the application Kubious HRV (version 2.0 Released November 2008). The interval selection of the rest seated conditions and during constant workload protocol was performed by visual inspection of the distribution of R-Ri (ms). The period with greater signal stability and sampling rate of 256 points as recommended by the *Task Force* ^22^, was selected. The analysis in the time domain was performed from the indices RMSSD (ms) - corresponding to the square root of the squared successive mean difference between adjacent R-Ri divided by the number of R-Ri less one and SDNN (ms) - standard deviation of all R-Ri .

### Statistical analysis

Data were subjected to a normality test (Shapiro-Wilk). As a non-normal distribution was observed, the non-parametric statistical tests were used. For comparisons between rest and exercise, the Wilcoxon test was applied, and for the conditions of exercise, the Kruskal-Wallis test with Dunn's post-hoc was used. For categorical variables, the Fisher exact test was applied. Analyses were performed with GraphPad Instat 3 software, with a significance level of p<0.05. Demographics, anthropometrics and clinical data were presented as means with standard deviation, and the variables related to the conditions of exercise were presented in median (maximum - minimum).

## Results

**Figure 1:**
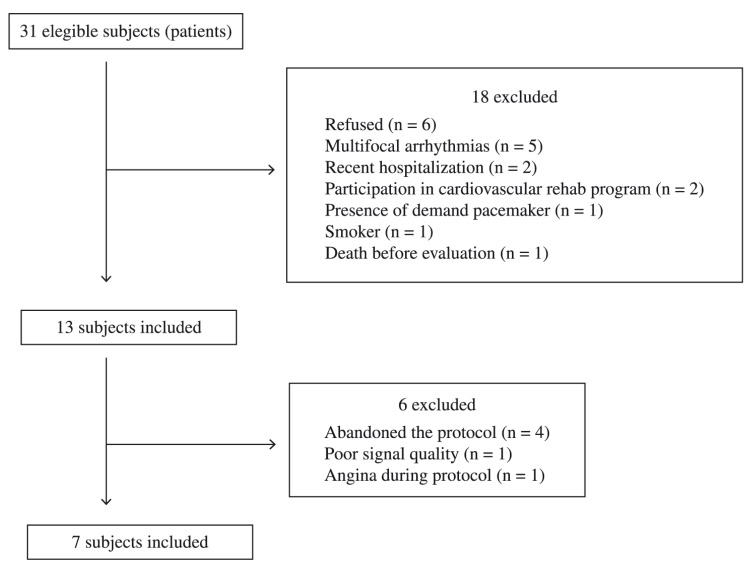
Flowchart of the study.

Initially, 31 subjects with CHF were screened. Twenty-four were excluded and only seven included in the survey, as shown in Figure 1.

Table 1 shows the anthropometric and clinical characteristics of the sample. The subjects had reduced left ventricular ejection fraction mostly of ischemic origin, with functional class II/III and preserved pulmonary function. Regarding BMI, individuals were eutrophic or slightly overweight. In the incremental test, the average load achieved by the subjects was 36 watts, with suppressed blood pressure and HR response. All subjects were on optimal medication and used β - blockers (mean dose of 42±11 mg).

**Table 1:** Anthropometrics and clinical characteristics of Chronic Heart Failure (CHF) subjects.

	CHF (n=7)
Age (years)	62±8
Height (m)	1.66±0.07
Mass (kg)	68.06±9.84
Body mass index (kg/m²)	24.67±3.73
**Echocardiography**	
Left ventricle ejection fraction (%)	41±8
**Etiology of CHF**	
Ischemic	4
Nonischemic	3
**NYHA class**	
II/III	4/3
**Spirometrics**	
FEV_1_ (% predict)	80.29±8.58
FEV_1_/FVC (%)	82.00±4.24
**Clinical characteristics **	
SpO_2 _(%)	96±2
RF (ipm)	14±4
**Drugs**	
Diuretics	3
Digitalis	5
b – blocker	7
Angiotensin-converting enzyme inhibitor	6
**Incremental test**	
At rest	
SAP (mmHg)	110±10
DAP (mmHg)	75±9
HR (bpm)	67±9
Peak	
SAP (mmHg)	141±18
DAP (mmHg)	80±10
HR (bpm)	100±20
Power (watts)	36±9

Values are means ± SD

FEV1 : forced expiratory volume in the first second

FEV1/FVC : forced expiratory volume in the first second and forced vital capacity ratio

SpO2 : peripheral oxygen saturation

RF : respiratory frequency

SAP : systolic arterial pressure

DAP : diastolic arterial pressure

HR : heart rate

NYHA : New York Heart Association

**Figure 2:**
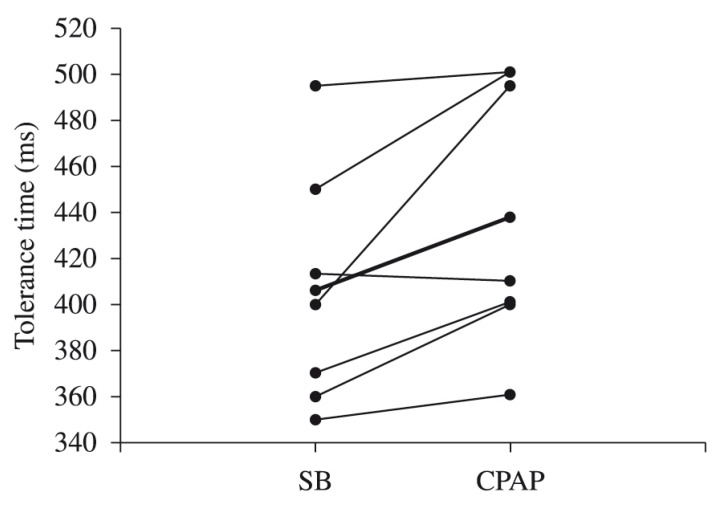
Tolerance time for Chronic Heart Failure Subjects during submaximal exercise at 75% of incremental test. SB: spontaneous breath. Median (dark line).

Table 2 shows the cardiorespiratory variables and the subjective exertion scale (Borg CR-10) for the conditions studied. As expected, there was a significant difference in HR and RR variables between rest and exercise conditions (p<0.05). Systolic blood pressure increased significantly during exercise conditions in 75% of the incremental protocol. With regard to the tolerance time, a statistically significant difference between the CPAP conditions and spontaneous respiration was observed during 75% of the intensity incremental test (Table 2 and Figure 2). In addition, HR showed higher values in the condition CPAP in relation to spontaneous breathing at this intensity (p<0.05). There were no significant differences in the other variables investigated.

**Table 2:** Cardiopulmonary variables and Borg scale during exercise for Chronic Heart Failure Subjects.

Variables	50% incremental test		75% incremental test	
	SB	CPAP	SB	CPAP
**At rest**				
RF (ipm)	14 (12-15)	13 (12-16)	14 (12-14)	14 (12-15)
HR (bpm)	71 (56-80)	67 (56-78)	68 (56-73)	78 (56-80)
SAP (mmHg)	110 (90-120)	100 (90-110)	110 (90-120)	110 (90-120)
DAP (mmHg)	75 (70-85)	75 (70-85)	70 (80-90)	75 (70-85)
**Submaximal peak-exercise**				
Tolerance time (s)	462 (315-505)	460 (360-503)	400 (350-495)	410 (361-501)^†^
HR (bpm)	99 (95-120)*	98 (94-114)*	97 (93-102)*	104 (103-108)*^†^
RF (ipm)	21 (18-22)*	20 (18-22)*	24 (22-26)*	23 (21-25)*
SAP (mmHg)	130 (125-135)	130 (120-135)	145 (130-150)*	150 (135-160)*
DAP (mmHg)	85 (70-90)	90 (70-95)	90 (70-95)	90 (70-95)
Dyspnea score	5(1-7)	5 (3-8)	5 (4-8)	5 (3-7)
Leg effort score	5 (2-7)	4 (2-7)	6 (3-8)	5 (3-7)

Median (min-max)

SB : spontaneous breath

CPAP : continuous positive airway pressure

RF : respiratory frequency in incursions to minutes

HR : heart rate in beat to minutes

SAP : systolic arterial pressure

DAP : diastolic arterial pressure

* p<0.05: rest vs. exercise (Wilcoxon test)

† p<0.05: SB vs. CPAP (Kruskall-Wallis test with Dunn's post-hoc).

**Figure 3:**
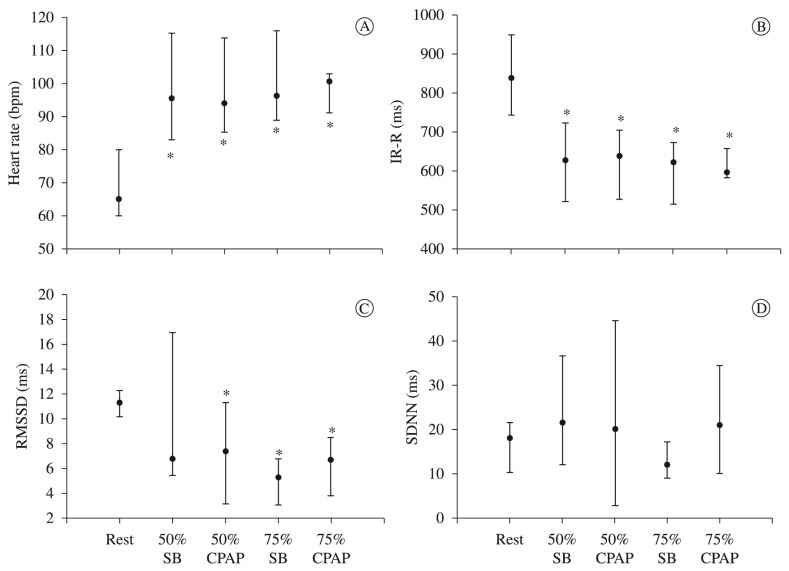
Heart rate variables of Chronic Heart Failure Subjects during testing. (A) heart rate in bpm; (B) R-R intervals in ms; (C) RMSSD index in ms; (D) SDNN in ms. *p<0.05. Kruskall-Wallis test with Dunn's post-hoc. IR-R: R-R intervals of electrocardiogram signals; RMSSD: The square root of the mean of the sum of the squares of differences between adjacent NN intervals; SDNN (ms): standard deviation of R-R intervals in ms; SB: spontaneous breath; CPAP: continuous positive airway pressure.

In the HRV analysis, HR was significantly higher during the physical exercise conditions, followed by a decrease in mean RR (p<0.05) intervals. While the SDNN index showed no statistical difference between the conditions studied, the RMSSD showed lower values in the conditions 50% with CPAP and 75% with and without CPAP, when compared to the rest condition (p<0.05) (Figure 3).

## Discussion

The main findings of the study were: (i) increased duration of subject exercise tolerance with CHF in loads of 75% with CPAP, (ii) there was no difference in HRV between the conditions of physical exercise studied, and finally, (iii) the RMSSD index (representative of vagal modulation) revealed significantly lower values during the physical exercise conditions when compared to rest.

At baseline, the subjects were mostly eutrophic, with left ventricular ejection fraction reduced at ischemic levels. All subjects had preserved lung function and showed reduced functional capacity presented by the lower peak load at the incremental test. In addition, all subjects kept optimal use of their medication, with β - blockers and angiotensin-converting enzyme the most frequent drugs in use. During the protocols of constant load, the subjects showed a significant increase in HR and RF at exercise compared to rest. These results were expected; although it may have been influenced by the health condition of the individuals, since human systems will always determine the onset of central and peripheral adjustments (by mechanisms of feeding/retro-feeding) in search of homeostasis before a new demand is imposed^16^ ^,^ ^23^.

The main finding of the current study was the increased time of physical exercise tolerance observed at 75% of the incremental load test with CPAP. Borghi-Silva et al.^7^ observed a significant difference on the time of exercise tolerance in patients with heart failure when subjected to proportional assisted ventilation with volumes and flows titrated of 5.7±1.5 H_2_O/l and 3.1±1.1 cmH_2_O.l^-1^.s^-1^, respectively, concomitantly to constant load exercise of high intensity (70-80% of peak load of the incremental test). In another study, Chermont et al.^8^ also observed an increase in exercise tolerance with an increase in the distance walked during the 6-minute walk test and chronotropic reserve of patients with CHF when submitted to CPAP - 6 cmH_2_O for 30 minutes before the test. In a similar research protocol, Lima et al.^24^ also found an increase in distance walked during the 6-minute walk test in patients with chronic heart failure when experiencing CPAP - 10 cmH_2_O prior to testing.

Dempsey et al.^1^ have shown that in high intensity exercise, blood flow redistribution occurs from the peripheral muscles to the ventilatory muscles (theory of shunt). This occurs due to the overload of ventilatory muscles that demand an increment of 30% of the relative cardiac output. As a result, there is less blood supply to peripheral muscles, which induces an early fatigue. Accordingly, the association of ventilatory support to physical exercise can facilitate the work of the respiratory musculature by decreasing the lower metabolic demand and permitting better redistribution of blood flow to the peripheral muscles^1^ - which might explain our findings. Likewise, the improvement in cardiac performance may also have been responsible for the increased exercise tolerance, since the application of CPAP reduces the transmural pressure of the left ventricle, improving cardiac output and reducing the end-systolic^25^. In this respect, our results on gains of physical exercise tolerance might be related to improvements in oxygen supply to peripheral muscles, at the expense of redistribution of blood flow even with CPAP of 5 cmH_2_O. We emphasize that the increase in tolerance time was followed by an increase in HR because the metabolic demand is high.

In addition to the above, we observed a reduction in the subjective effort scale (Borg CR-10) between the conditions RE and CPAP during physical exercise of constant load. Although the data showed no statistical difference, some studies^26^ ^,^ ^27^ have shown that a two-point change on the Borg scale is clinically important. This finding confirms our hypothesis that blood flow was more evenly distributed in the peripheral muscles in condition CPAP, which has led to a greater tolerance to physical exercise.

Regarding HRV, previous studies^28^ ^,^ ^29^ have shown that patients with CHF have sympathetic-vagal imbalance at rest and this can be mitigated by proper titration pressure level during the application of CPAP at rest. However, our results revealed no differences between the values of SDNN index (representative of the total HRV) under the conditions of physical exercise studied. These findings support the hypothesis that the application of CPAP pressure level of 5 cmH_2_0 did not bring major hemodynamic consequences, since the increase in intra-thoracic pressure (through positive pressure) can result in significant hemodynamic changes^30^. Although, we used a different exercise protocol and modality of NIV, our data corroborated with Borghi-Silva et al.'s^7^ findings that the effect of proportional assisted ventilation during physical exercise on cardiac output variables, systolic volume and HR, can be assessed noninvasively by thoracic bioimpedance. The authors showed that patients with CHF exhibited improvement in physical exercise tolerance during application of non-invasive ventilation without significant changes in hemodynamic variables.

Concomitantly, the HRV analysis showed a decrease in the mean iR-R with an increase in mean HR during physical exercise compared to the resting condition. This finding can be easily explained by the increased metabolic demand during the transition from rest to physical exercise^23^ ^-^ ^31^, which is present in individuals with chronic cardiopulmonary disorders, even if influenced by the patient's condition. Interestingly, these data were followed by a decrease in the RMSSD index (representative of vagal modulation). This implies that the adjustment of HR to exercise may be due to a lower parasympathetic modulation, since these individuals were using β-blockers - the type of drugs that are known to influence the sympathetic modulation on the control of HR^32^.

### Study limitations

A larger sample size of CHF patients would be desirable. However, the smaller sample size of this study is attributed to the complexity of CHF disease that influenced sample size loss during the research. Another aspect relates to the influence of the use of β - blockers on HRV analysis, but currently this is the therapy of choice to preserve cardiac function and myocardial metabolic demand in patients with CHF.

### Clinical implication

As presented in our study, patients with CHF had a reduced exercise tolerance and appeared to benefit from NIV application associated with physical exercise. Therefore, this might be a good strategy for the treatment of patients to improve their functional capacity in cardiovascular therapy programs.

## Conclusion

Our study showed improved functional capacity of patients with CHF when submitted to CPAP at loads of 75% of incremental test, but no improvements in exercise tolerance at loads of 50% of incremental test. Additionally, no significant change in HRV during the exercise conditions studied was observed. Moreover, decreased values of RMSSD index (representative of vagal modulation) were observed in the transition from rest to physical exercise, suggesting the adjustment of the HR was predominantly sympathetic and at the expense of reduced parasympathetic modulation when submitted to NIV.
